# Reporting of diagnostic and laboratory tests by general hospitals as an indication of access to diagnostic laboratory services in Kenya

**DOI:** 10.1371/journal.pone.0266667

**Published:** 2022-04-08

**Authors:** Felix Bahati, Jacob Mcknight, Fatihiya Swaleh, Rose Malaba, Lilian Karimi, Musa Ramadhan, Peter Kibet Kiptim, Emelda A. Okiro, Mike English

**Affiliations:** 1 Health Services Research Unit, KEMRI Wellcome Trust Research Programme, Nairobi, Kenya; 2 Oxford Centre for Global Health Research, Nuffield Department of Medicine, University of Oxford, Oxford, United Kingdom; 3 Ministry of Health, Mama Lucy Kibaki Hospital, Nairobi, Kenya; 4 Ministry of Health, Kakamega County Referral Hospital, Kakamega, Kenya; 5 Ministry of Health, Meru county, Meru, Kenya; 6 Ministry of Health, Nakuru Provincial General Hospital, Nakuru, Kenya; 7 Ministry of Health, Kenyatta National Hospital, Nairobi, Kenya; 8 Population Health Unit, KEMRI-Wellcome Trust Research Programme, Nairobi, Kenya; Public Library of Science, UNITED KINGDOM

## Abstract

**Introduction:**

Information on laboratory test availability and current testing scope among general hospitals in Kenya is not readily available. We sought to explore the reporting trends and test availability within clinical laboratories in Kenya over a 24-months period through analysis of the laboratory data reported in the District Health Information System (DHIS2).

**Methods:**

Monthly hospital laboratory testing data were extracted from the Kenyan DHIS2 between January 2018 and December 2019. We used the national laboratory testing summary tool (MoH 706) to identify the tests of interest among 204 general hospitals in Kenya. A local practitioner panel consisting of individuals with laboratory expertise was used to classify the tests as common and uncommon. We compared the tests on the MoH 706 template with the Essential Diagnostic List (EDL) of the World Health Organisation and further reclassified them into test categories based on the EDL for generalisability of our findings. Evaluation of the number of monthly test types reported in each facility and the largest number of tests ever reported in any of the 24 months were used to assess test availability and testing scope, respectively.

**Results:**

Out of the 204 general hospitals assessed, 179 (179/204) reported at least one of the 80 tests of interest in any of the 24 months. Only 41% (74/179) of the reporting hospitals submitted all their monthly DHIS2 laboratory reports for the entire 24 months. The median testing capacity across the hospitals was 40% with a wide variation in testing scope from one hospital laboratory to another (% IQR: 33.8–51.9). Testing scope was inconsistent within facilities as indicated by often large monthly fluctuations in the total number of recommended and EDL tests reported. Tests of anatomical pathology and cancer were the least reported with 4 counties’ hospitals not reporting any cancer or anatomical pathology tests for the entire 24 months.

**Conclusion:**

The current reporting of laboratory testing information in DHIS2 is poor. Monitoring access and utilisation of laboratory testing across the country would require significant improvements in consistency and coverage of routine laboratory test reporting in DHIS2. Nonetheless, the available data suggest unequal and intermittent population access to laboratory testing provided by general hospitals in Kenya.

## Introduction

Laboratory diagnosis is essential to the quality of care offered in any health care system [1]. Clinical laboratory tests are useful for disease surveillance, disease detection, guiding treatment, prognosis and making referrals [2]. Evidence shows that 60–70% of clinical decisions depend on laboratory diagnosis [3, 4]. Delays in diagnosis linked to inadequate diagnostic capacity also compromise patient outcomes and increase hospital length of stay [2, 5]. More generally, diagnostic testing is critical to care for patients with chronic diseases at the emergency department [5], for primary healthcare providers helping them avoid empirical therapies and for early detection of infectious diseases such as HIV, TB and COVID-19 to minimize spread [6].

The World Health Organization (WHO) advocates for clinical laboratory testing across all tiers of healthcare systems worldwide [7]. This was partly addressed by the development of an Essential Diagnostic List (EDL) consisting of 122 clinical diagnostic tests [7]. The tests are essential because they satisfy most of the population’s health care needs, especially when customized according to regional disease prevalence and resource availability. The tests also provide a good focus for plans to achieve Universal Health Coverage (UHC) [6] as they are cost-effective, relevant to public health and highly utilized. A subset of these tests is especially helpful in supporting primary and first-level referral healthcare in Low and Middle-Income Countries (LMICs) [2, 8].

Access to laboratory and pathology diagnostic services in LMICs remains a challenge [1]. Several surveys conducted on the availability of essential diagnostic tests in LMICs have shown a crucial gap especially in public facilities [9, 10]. Besides the low test availability, reports have shown a lot of variations in the scope of essential tests offered across hospital tiers in LMICs [11]. A study assessing the availability of some essential diagnostic tests within ten LMICs observed an overall test availability of 19.1% for basic primary care, 49.2% for advanced primary care and 68.4% among hospitals [12]. Specifically, Kenya recorded a test availability of 55.5% across the health facilities sampled in this survey [12]. The ability to offer clinical laboratory testing beyond basic diagnostics such as malaria smears in Kenya is largely concentrated in the public sector at level 4 and 5 facilities (county hospitals). It is to these facilities that patients in need of diagnostic tests would often be referred from primary care [13]. Therefore, these hospitals are expected to have well-functioning laboratories able to meet diagnostic needs across all ages especially as the country strives to meet the Sustainable Development Goals (SDGs) and provide care for non-communicable diseases (NCDs) and common cancers.

Although the diagnostic expectations of clinical laboratories in Kenya are articulated, there is a paucity of data on the current scope and capacity of these clinical laboratories. The Ministry of Health (MoH) conducted a Service Availability and Readiness Assessment (SARA) among Kenyan health facilities [14]. However, the survey mostly provided information on laboratory equipment and personnel, making it difficult to deduce the actual availability of tests offered in these clinical laboratories. Separately, the MoH aims to collect monthly data from hospitals in Kenya through the District Health Information System (DHIS2) [15]. Hospital laboratory testing activities are reported in the DHIS2, thus providing an opportunity to monitor coverage and availability of laboratory testing across the country. Here, we explore the reporting trends of hospitals’ clinical laboratories in Kenya over a 24-months period, between January 2018 to December 2019.

## Materials and methods

### (a) Hospital selection

We sought to understand laboratory test availability in Kenyan hospitals through laboratory reports submitted in DHIS2. We focused on a set of 201 hospitals in Kenya identified by Ouma et al using different data sources on health services availability from government health facility surveys and routine inpatient admission data [16]. The hospitals were identified as meeting 6 criteria. These included; availability of an operating theatre, at least two medical officers and reported conduct of caesarean sections, x-ray, oxygen support, neonatal incubators and blood transfusion services [16]. We reasoned hospitals offering these services should have functioning hospital laboratories. We updated this list to include 3 more hospitals meeting the same criteria making the total number of eligible hospitals studied 204. These hospitals represent about 51% (204/399) of all the hospitals in Kenya [16] and we later categorized them as urban, peri-urban and rural hospitals based on their locality. We complemented these data by obtaining the number of beds in each of the 204 hospitals, retrieving these data on bed capacity from the Kenya Master Health Facility List (KMHFL) [17]. The bed capacity in these 204 facilities ranged from 8–1455.

### (b) Identification of the clinical laboratory tests for analysis

Our analyses were based on clinical laboratory reports that should be captured in a national laboratory testing summary tool (MoH 706) designed by the Kenyan government [18]. It should be used by all clinical laboratories in Kenya to aggregate monthly testing information before data are uploaded into the DHIS2 platform. The tool encompasses 91 tests organised into 9 categories: urine analysis, blood chemistry, parasitology, haematology, bacteriology, histology and cytology, serology, specimens referred to higher levels and drug susceptibility testing. We did not include the drug susceptibility test category in our analyses as laboratories do not report on the exact number or variety of tests performed; the tool operates only to promote reporting of specific sensitive and resistant samples. We also excluded the *Yersinia pestis* test from further consideration as none of the 204 hospitals had ever reported conducting the test. Similarly, four blood screening tests (HIV, Syphilis, Hepatitis C and Hepatitis B) were also excluded because these tests are conducted at a much smaller number of blood donation satellites and not within hospitals’ clinical laboratories. Finally, although the MoH 706 tool also incorporates reporting of bacterial assessment on water and food, these tests were not included in the set of test reports we analysed as our interest is clinical tests supporting patient care.

A total of 80 of the 91 possible tests were therefore included in our analyses. Although these tests are divided into 9 test categories in Kenyan reports, we reclassified them into the 6 categories defined in the World Health Organization’s Essential Diagnostic List (WHO EDL) to make our findings more generalisable. These categories are; clinical chemistry, haematology, anatomical pathology, cancer, sexually transmitted infections and bacteriology, mycology and parasitology. Of the 80 tests we examined, 46 are directly named in the WHO EDL as shown in **Table 1**, the remaining Kenyan tests were allocated to WHO EDL categories by the authors who include individuals with laboratory expertise.

**Table 1:** Test categories of the 80 laboratory tests analysed.

Test categories	Test number (N = 80)		EDL Tests (N = 46)		Definitions
**Clinical chemistry**	**16**	C = 13 U = 3	**13**	C = 12 U = 1	Tests involving biochemical analysis of body fluids especially blood. Examples include; blood sugar tests, Liver Function Tests among others.
**Cancer**	**3**	C = 1 U = 2	**1**	C = 1 U = 0	Cancer detection tests such as Prostate Specific Antigen Test (PSA).
**Anatomical pathology**	**24**	C = 5 U = 19	**-**	** -**	Consists of histology and cytology tests
**Sexually Transmitted Infections**	**5**	C = 4 U = 1	**4**	C = 3 U = 1	Tests to diagnose sexually transmitted infections such as HIV, Syphilis among others.
**Hematology**	**8**	C = 6 U = 2	**7**	C = 6 U = 1	Tests to diagnose blood related abnormalities such as infections, inflammations, anemia etc.
**Bacteriology, Mycology & Parasitology**	**24**	C = 18 U = 6	**21**	C = 16 U = 5	Tests to determine the presence or absence of bacteria, fungi or other parasites.

N/B: The classification of the Common (C) and the Uncommon (U) tests illustrated in this table has been explained in the methods below.

Laboratory testing data were extracted from the DHIS2 for a duration of 24 months; from January 2018 to December 2019. We used the MoH 706 template to identify the tests of interest among the 204 hospitals and exported data as a CSV file. Initial examination of the exported data revealed many missing values. We compared original paper copies of laboratory data on the MoH 706 form with the DHIS2 data from two hospitals to explore the reasons for missing data. This revealed that values recorded as missing in the DHSI2 database had initially been captured as zero values on the paper copies of the filled MoH 706 tool. Discussions with Health Records Information Officers (HRIOs) and laboratory managers clarified that the DHIS2 system converts zero values entered at hospital level to missing values (N/A) [19], a feature of DHIS2 that applies also to clinical data [20]. To account for this, we assumed that any missing value in the dataset was in fact a zero value, implying that the hospital’s laboratory did not conduct the test in that month. If the retrieved DHIS2 data showed missing / zero test volumes for all the 80 tests of interest, we assumed this was a truly missing report from a hospital for that month.

### (c) Analysed variables

The reporting frequencies of the 204 hospitals were first assessed, which helped identify the non-reporting and reporting hospitals. A hospital was classified as non-reporting if there were no reports for all 80 tests throughout the 24 months studied. Reporting hospitals were hospitals that had reported a test volume of greater than zero for any test in any of the 24 months.

We used a local practitioner panel of 5 laboratory managers (also co-authors) to classify the 80 tests of interest into two broad categories; common and uncommon tests (Table 2). This classification was necessary because uncommon diagnostic tests would rarely be requested by clinicians or doctors in one month. Consequently, it would be justifiable for a hospital to report zero activity for several months for such uncommon tests within the 24 months analysed especially in smaller facilities. Common tests are frequently conducted in hospital laboratories and, according to the practitioner panel, it is reasonable therefore to expect that reports for these tests each month would not be zero if the laboratory is functioning well. This classification resulted in 47 common tests and 33 uncommon tests (**Tables 1** and **2**). We confirmed that all the 47 common tests were reported for at least 18 of the 24 months in some of the hospitals. Equally, all the 33 uncommon tests had non-zero test volumes for at least some months among the 204 facilities.

**Table 2:** Showing variable definitions.

Variable	Definitions
**Reporting hospitals**	Hospitals that reported a test volume of greater than zero for any test in any of the 24 months.
**Non-reporting hospitals**	Hospitals that did not report a test volume of greater than zero for any test within the 24 months duration.
**Common Tests**	Diagnostic tests that are frequently conducted in clinical laboratories.
**Uncommon Tests**	Diagnostic tests that are rarely conducted in clinical laboratories such that sometimes a month would elapse without them being conducted.
**EDL**	An Essential Diagnostic List of tests as published by the WHO.
**TTT (Total Test Types)**	The largest scope of tests ever reported in any of the 24 months within a hospital.

Using hospitals’ monthly reports, we were able to calculate the number of types of tests that the hospitals reported each month (with a highest possible value = 80). We used simple line plots of the number of test types reported each month to visually explore hospitals’ reporting patterns represented by the fluctuation in the number of test types reported. Moreover, each hospitals’ reports were used to calculate the largest number of test types it ever reported in any of the 24 months within a facility, we termed this a hospital’s Total Test Types (TTT). The TTT thus represents the highest possible number of tests out of all the 80 tests a hospital seems able to conduct. Consequently, the TTT may be used to explore variation in capacity across hospitals’ laboratories when they are operating to their full potential. For instance, we used the TTT to explore how the testing scope varied using hospital bed capacity as an indicator of hospital size [21]. We categorized the number of beds in each hospital into 4 categories (<100 beds, 100–199, 200–299 and 300+ beds) and then plotted the TTT against these categories to reveal the trends.

Finally, we specifically investigated the availability of cancer and anatomical pathology tests within Kenya’s 47 counties. We grouped all the 179 reporting facilities by county and then examined if any of the hospitals reported any cancer or anatomical pathology tests of interest. All the unique cancer and anatomical pathology tests reported among all hospitals within a given county were counted. This helped us to understand the potential accessibility of both cancer and anatomical pathology tests across the counties.

### Ethical clearance

The data presented in this manuscript is part of the Clinical Information Network (CIN) study that was approved by KEMRI Scientific and Ethical Review Committee (3459). The Kenya Ministry of Health gave permission for this work which entailed use of anonymised routine patient care data.

## Results

### Hospitals and test characteristics

**Fig 1:**
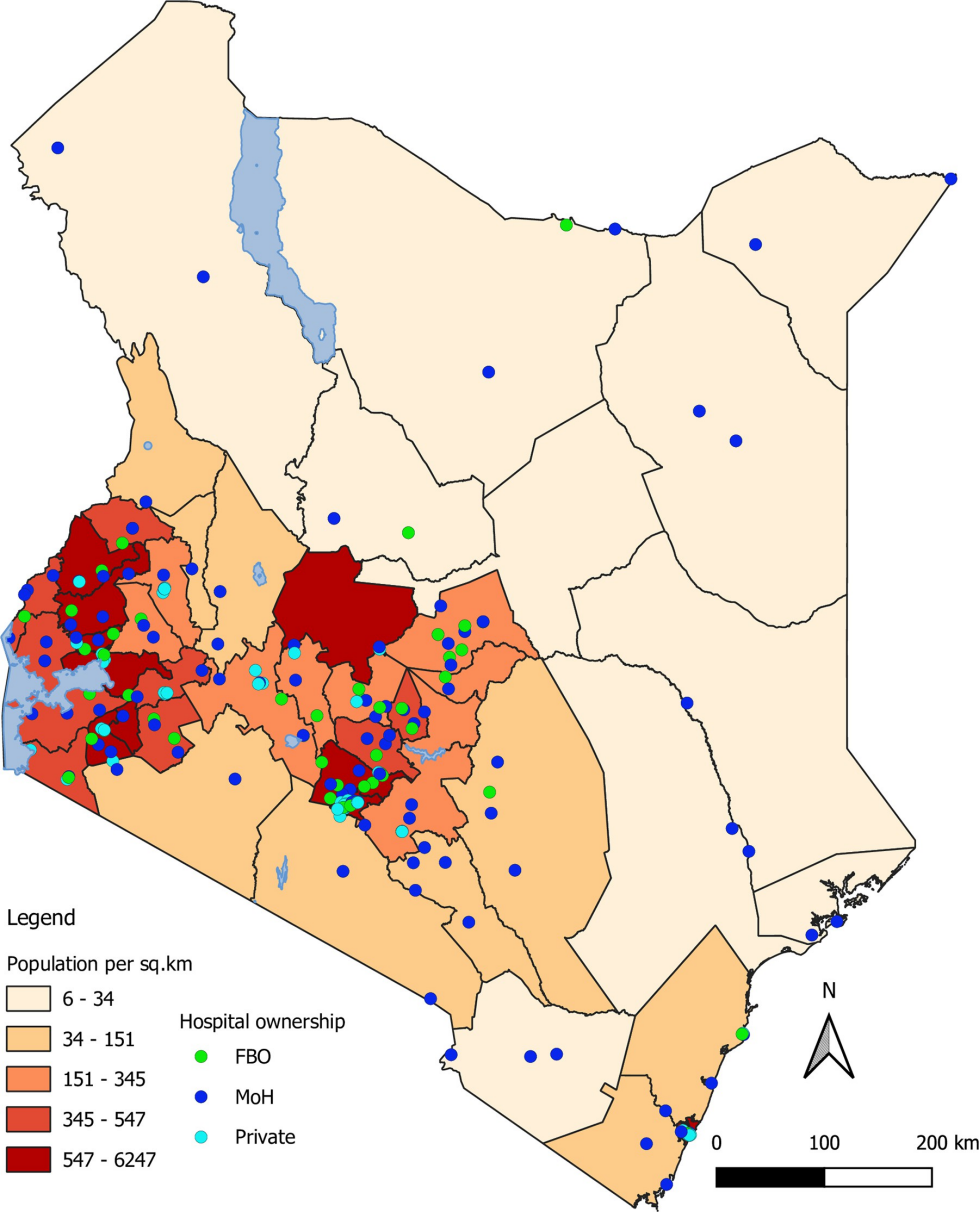
Distribution of the 204 hospitals across Kenya with the corresponding county population density based on 2019 census report in Kenya.

A total of 204 health facilities were initially selected for this analysis, of which 53.4% (109) were MoH, 25% (51) private and 21.6% (44) were Faith Based Organisations (FBO). According to the MoH hospital-level classification, 2 of the 204 facilities were level 3 facilities (the smallest inpatient facilities), 182 were level 4 (county and sub-county general hospitals), 18 were level 5 (larger county hospitals that were formally regional hospitals) and two were level 6 (tertiary hospitals) facilities. The distribution of these 204 health facilities across Kenya is shown in Fig 1 where 142 of them were in urban areas, 48 in peri-urban and 14 in rural areas. Out of these, 25/204 (12.3%; 3 FBO, 5 MoH and 17 Private) did not submit any laboratory data in DHIS2 for 24 months, and we excluded them from subsequent analyses. Neither of the two tertiary hospitals at level 6 submitted laboratory testing data in DHIS2. None of the 3 large private level 5 facilities submitted monthly laboratory reports in DHIS2.

The findings reported are therefore based on 179 hospitals (level 3, level 4 and level 5) of which 104 are MoH, 34 private and 41 FBO. The total number of laboratory tests expected to be reported was 80, of which 47 were classified as common tests while 33 were uncommon (S1 Table). Slightly more than half (57.5%, n = 46) of the 80 tests have been directly named in the WHO EDL. The number of test types reported per hospital across all months ranged from 0, representing no apparent laboratory activity across the 80 tests in a month, to 65 representing the maximum number of the 80 tests reported by any facility in the 24 months studied.

### Reporting patterns

**Fig 2:**
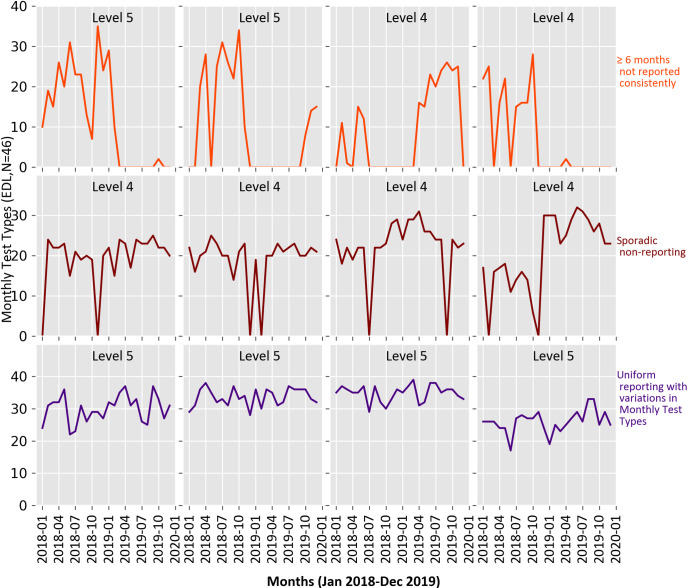
Monthly DHIS2 reporting trends of EDL tests in the year 2018 and 2019. The 4 facilities presented in each category were selected randomly.

Only 41.3% (74/179) of the reporting hospitals submitted monthly DHIS2 reports for the entire 24 months. Within such hospitals, the number of monthly test types reported fluctuated but in no month was the total number of test types zero. Of the 105 (105/179) facilities that did not report for all the 24 months, 23% (24/105) of them had periods of at least 6 months consecutively of non-reporting while 44% (46/105) were noted to have more sporadic non-reporting patterns. In the remainder (33%) of the 105 facilities, the number of months not reported consecutively ranged between 2 and 5. We illustrate the reporting variability and these patterns using the count of EDL test types (maximum = 46) for which activity was reported (Fig 2).

Analysis of the individual test reporting patterns across the six test categories was also conducted. Within each test category we sub-divided specific tests according to whether they were common or uncommon tests. A total of 165 hospitals (165/179) reported at least 1 month of activity (test volume >0) for any of the 33 uncommon tests while all the 179 reported activity for at least one of the 47 common tests. The tests that hospitals seemed most frequently able to offer across all the test categories were; *Helicobacter pylori*, blood sugar, Venereal Disease Research Laboratory (VDRL), Human Chorionic Gonadotropin (HCG), and rheumatoid factor which were offered at some point in all the 179 facilities. Other reporting patterns in each of the test categories are as described below.

#### Anatomical pathology

**Fig 3:**
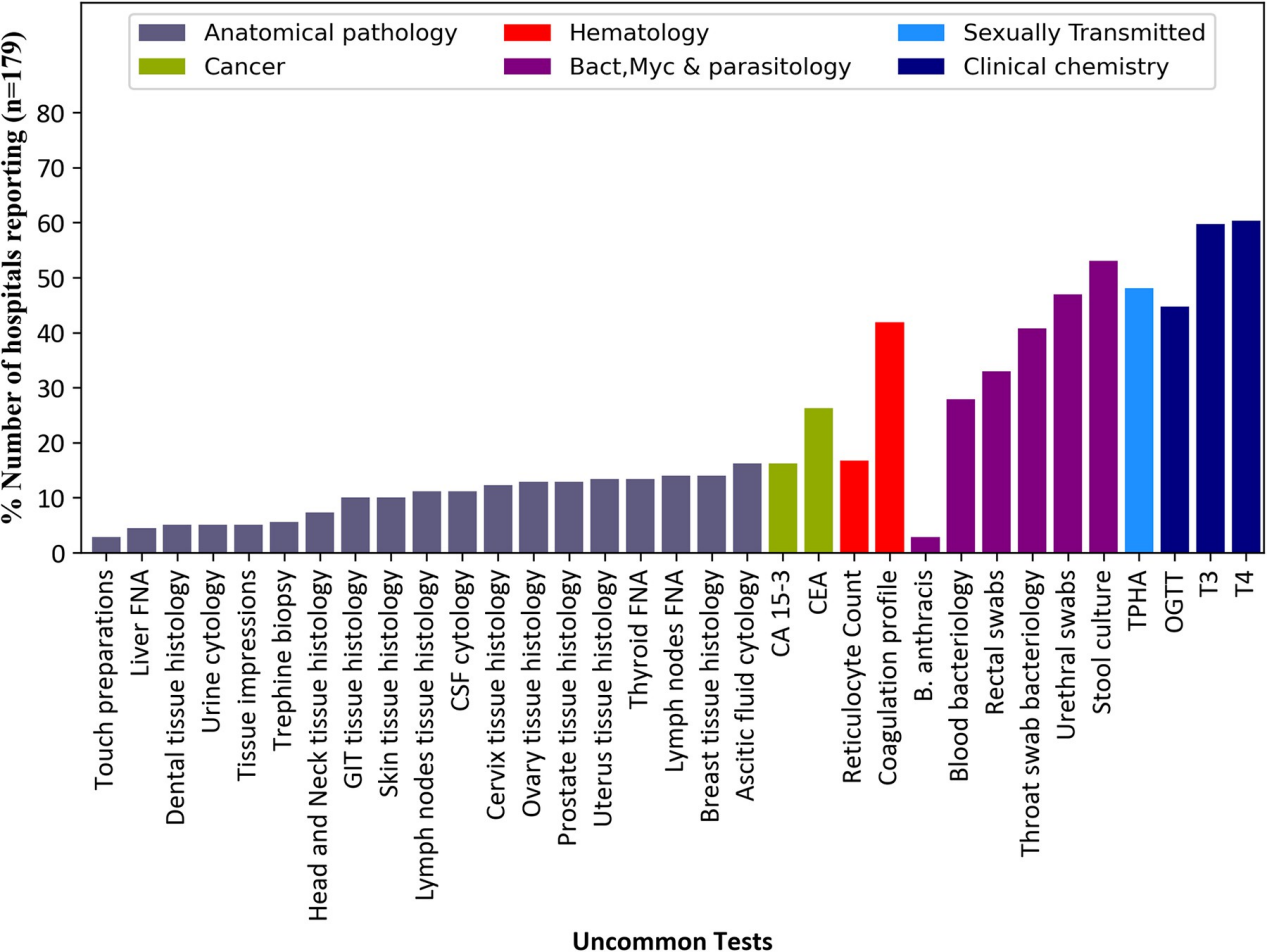
The reporting frequency of the 33 uncommon tests among 179 hospitals submitting data in DHIS2 for the years 2018 and 2019. All uncommon tests reported for at least one month were considered.

We examined 24 anatomical pathology tests, of which 5 were common while 19 were uncommon tests (**Table 1**). Generally, reports suggested limited access to anatomical pathology testing with none of the 24 tests reported in 25% or more of the reporting hospitals. The most reported uncommon tests within this category were; ascitic fluid cytology, breast tissue histology and Fine Needle Aspiration (FNA) of lymph nodes. Although the ascitic fluid cytology test had the highest reporting frequency, only 29/179 (16.2%) of the facilities ever reported performing this test. Equally, 86% (154/179) of the hospitals did not report on breast tissue histology and Fine Needle Aspiration (FNA) of lymph nodes test for breast cancer (Fig 3).

**Fig 4:**
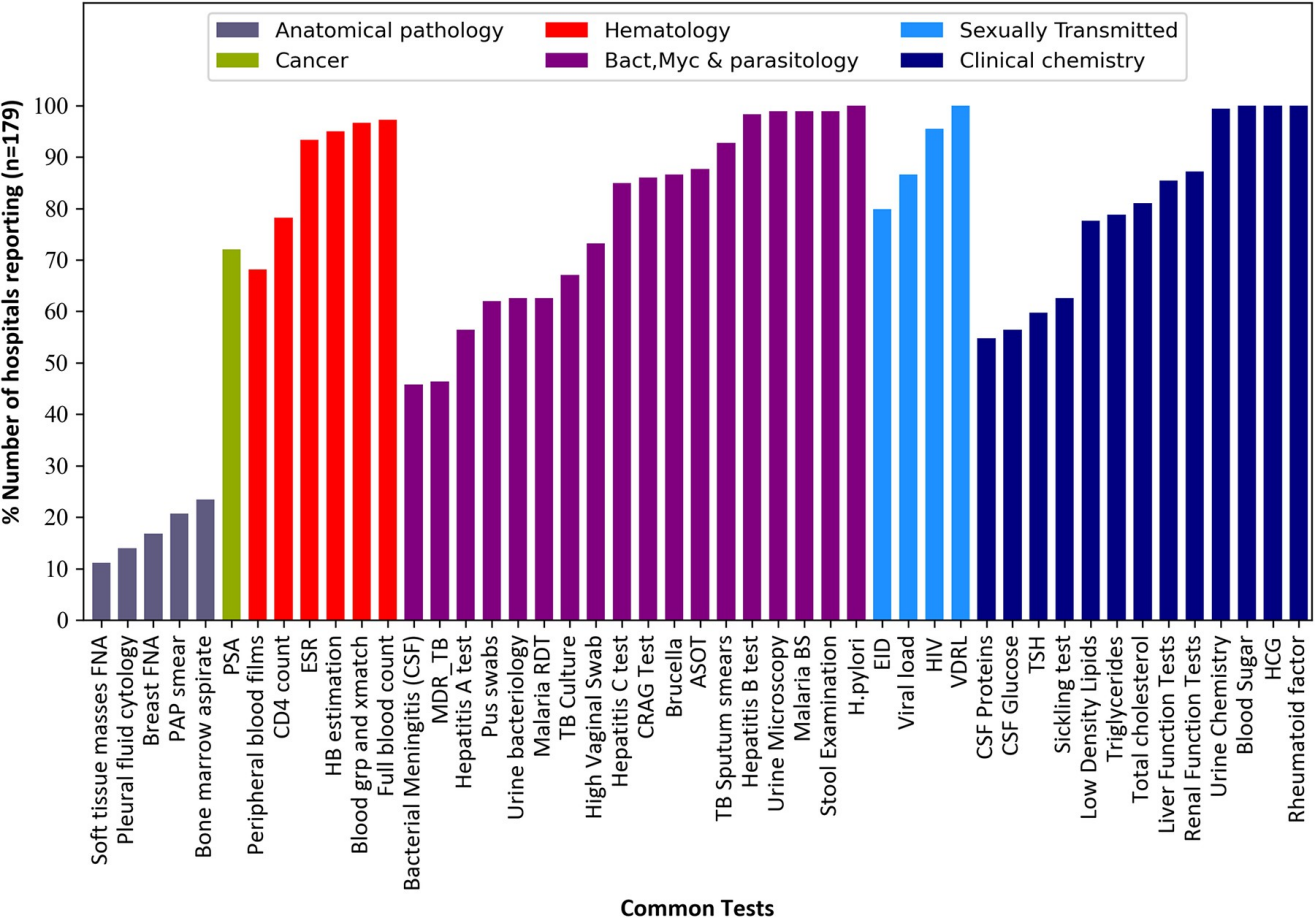
The reporting frequency of the 47 common tests among 179 hospitals submitting data in DHIS2 for the years 2018 and 2019. All common tests reported for at least one month were considered.

The 5 common anatomical pathology tests included: bone marrow aspirate, pleural fluid cytology, Fine Needle Aspiration of soft tissue masses (soft tissue masses FNA), Fine Needle Aspiration of the breast (breast FNA) and pap smear. The ability of hospitals to offer these tests is indicated in Fig 4 but Pap smear tests for cervical cancer screening and Fine Needle Aspiration of the breast for breast cancer were only reported by 20.7% and 16.8% of the facilities, respectively.

#### Cancer tests

Only 3 cancer antigen tests were explored in this analysis of which 2 were classified as uncommon while 1 was a common test (**Table 1**). The 2 uncommon tests were; Carcinoembryonic Antigen (CEA) and CA 15–3. A total of 73.7% (150/179) and 83.8% (132/179) of the hospitals failed to report any activity on CEA and CA 15–3 respectively, (CEA for colon cancer detection while CA 15–3 for monitoring prognosis of breast cancer therapy). The Prostate Specific Antigen (PSA) was the only common cancer antigen test and 72.1% (129/179) of the facilities offered this test at some point in 24 months (Figs 3 and 4).

### Haematology

We assessed a total of 8 haematology tests and out of these, 6 were common while 2 were uncommon tests (**Table 1**). The two uncommon tests were coagulation profile and reticulocyte count, and half of the facilities did not report any activity for either of these tests. Haematology tests of Full blood count and blood grouping and crossmatch were the most frequently reported common tests in this category, 97.2% and 96.6% of hospitals reported some activity conducting these tests respectively (Figs 3 and 4).

#### Bacteriology, mycology and parasitology

There were 24 tests of bacteriology, mycology and parasitology in this category. Of the 24 tests, 18 were common while 6 were uncommon tests (**Table 1**). The common tests that seemed mostly offered by hospitals were stool examination (98.9%) and testing for *Helicobacter pylori* (100%). Stool culture and urethral swabs were the most reported uncommon tests in this category, but activity was only reported in 53.7% and 46.9% of the facilities respectively, (Figs 3 and 4).

#### Sexually transmitted infections

Out of the 5 tests of sexually transmitted infections explored, 4 were common while 1 was uncommon test (**Table 1**). The *Treponema pallidum* hemagglutination test (TPHA) used in syphilis diagnosis was the only uncommon test and 48% of the facilities reported conducting it within the 24 months. The common tests VDRL (100%) and HIV (95.5%) were reported as conducted by a majority of the facilities (Figs 3 and 4).

#### Clinical chemistry

There were 16 tests of clinical chemistry of which 13 were common while 3 were uncommon (**Table 1**). The triiodothyronine (T3) and thyroxin (T4) hormonal tests of thyroid function were the most reported uncommon tests of clinical chemistry, 59.8% and 60.3% respectively. Of the 13 common tests in this category, blood sugar, HCG and rheumatoid factor were the most reported tests with all the 179 reporting facilities having reported them within any of the 24 months (Figs 3 and 4).

### Laboratory testing scope

**Fig 5:**
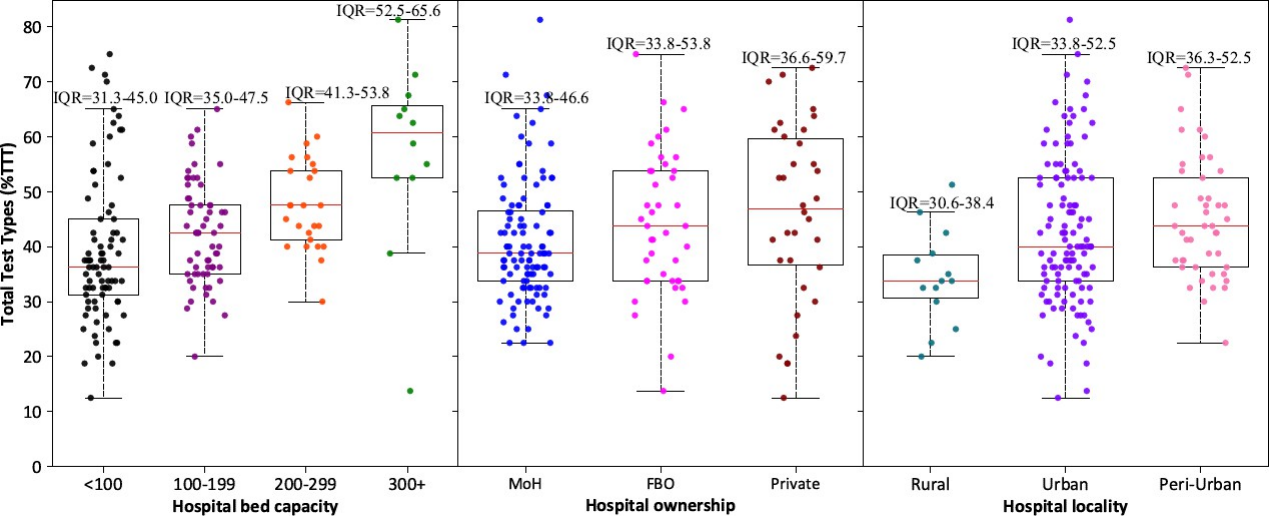
Testing scope in relation to the hospital bed capacity, hospital ownership and locality among general hospitals in Kenya based on DHIS2 laboratory data of the years 2018 and 2019.

Hospital bed capacity was compared with the highest number of tests (TTT) reported by each hospital within the 24 months. Of the 179 reporting facilities, 81 had less than 100-bed capacity, 61 had 100–199 beds, 25 had 200–299 and only 12 had 300 beds and above. The median number of TTT increased with an increase in hospital bed capacity: 36.3% in less than 100-bed capacity hospitals, 42.5% among 100–199 bed capacity, 47.5% in the 200–299 and 60.6% of tests among the 300+ bed capacity. Equally, the highest TTT (81%; 65/80) was noted among hospitals with 300 beds and above. We observed a lot of variation in testing scope among the smaller facilities of less than 100-bed capacity (Fig 5).

Stratification of TTT by hospital ownership showed a difference in testing capacities of these hospitals. The median testing capacity across the three types of hospitals was 40% (IQR: 33.8–51.9). The lowest testing capacity was noted among public facility laboratories (38.8%) while private hospital laboratories had the highest testing capacity (46.9%). Notably, there was a huge variation in the testing scopes of the private hospital laboratories (%IQR: 36.6–59.7) compared to FBO (%IQR: 33.8–53.8) and public hospitals (%IQR: 33.8–46.4) as presented in Fig 5. Similarly, testing capacity varied across hospital laboratories in rural, peri-urban and urban areas. The highest median testing capacity was observed among hospital laboratories in peri-urban areas (43.8%), followed by urban (40%) and rural (33.8%) laboratories. Hospital laboratories in urban areas, however, had the highest variations in testing scope (%IQR: 33.8–52.5) as illustrated in in Fig 5.

**Fig 6:**
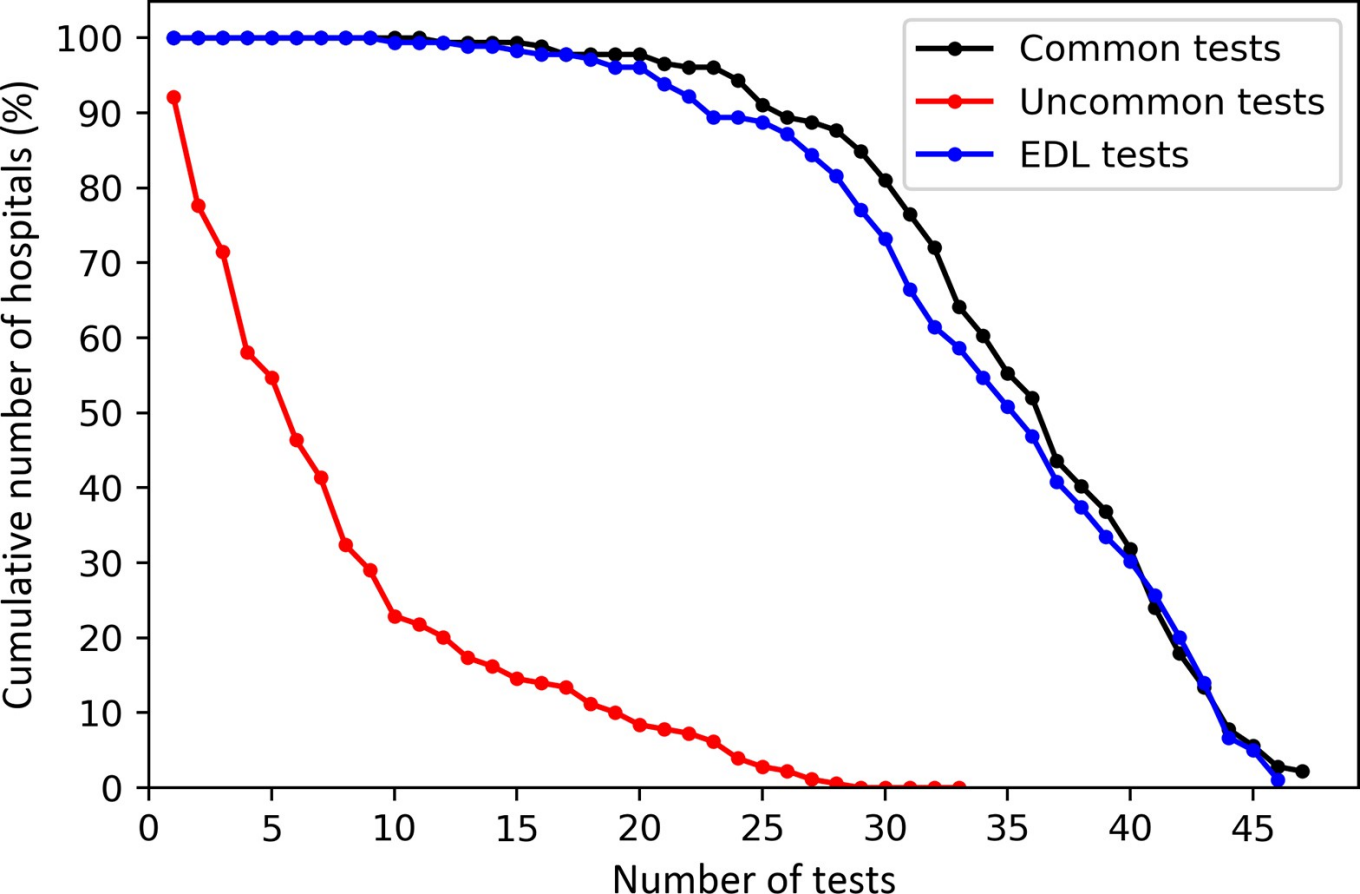
Variation in testing scope of common (n = 47), uncommon (n = 33) and EDL (n = 46) tests reported in any of the 24 months. All the 179 facilities reported common and EDL tests while 165 reported uncommon tests. Cumulative hospital percentages were calculated based on the 179 reporting facilities.

Plotting the cumulative number of hospitals against the testing activities revealed a wide variation in the laboratory testing scope. We observed that 90% of the 165 hospitals reported no testing activity for at least 19 (19/33) of the uncommon tests. Similarly, 19% of the 179 facilities reported activity for fewer than 30 of the 47 common tests over the 24 months. Focusing on the EDL tests, 27% of the 179 facilities reported performing a maximum of 30 (30/46) of these tests at any time over the two years (Fig 6).

### Availability of cancer and anatomical pathology tests within Kenya’s 47 counties

**Fig 7:**
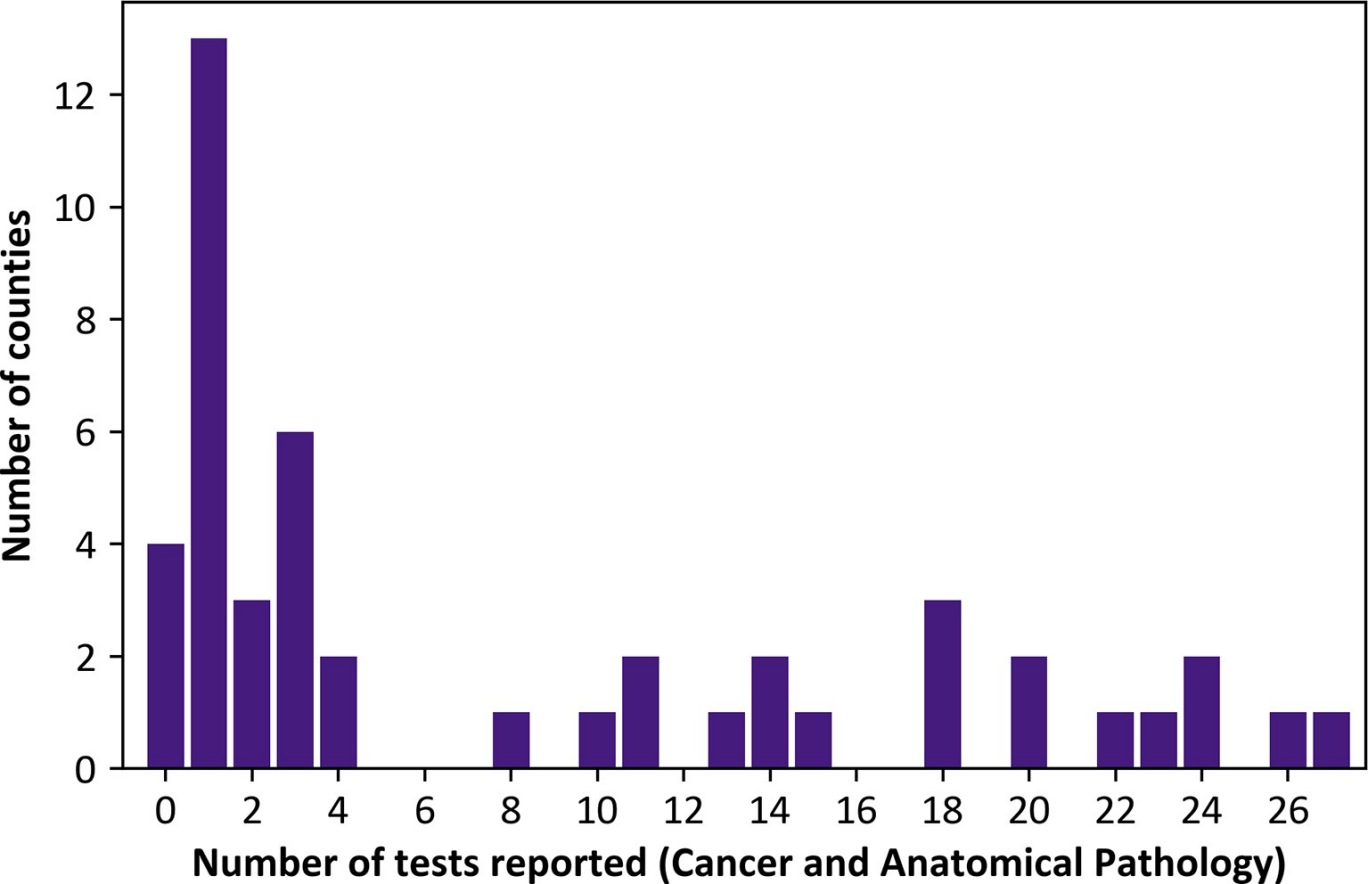
Number of cancer and anatomical pathology tests ever reported within county hospitals in the year 2018 and 2019.

The total number of cancer and anatomical pathology tests assessed for availability was 27. Out of the 47 counties, 4 of them reported no cancer nor anatomical pathology testing activities for the 24 months analysed and many counties (27.7%, 13/47) reported offering only one of the 27 anatomical pathology and cancer tests examined at any point over 24 months (Fig 7). Only 8 (8/47) counties reported at least once conducting 20 of the 27 tests with 1 of them reporting at least some activity for all the 27 tests assessed.

## Discussion

The desire for better Hospital-based routine data in LMICs is not new [22], and DHIS2 offers a mechanism through which such data are captured. The DHIS2 can provide robust routine data that could be useful in monitoring health services provision, including laboratory testing across health systems [23]. Monthly hospital laboratory testing data are aggregated and reported by laboratory managers and HRIOs. Yet, it remains unclear whether the reported data serve the intended purpose. Any relevant data that is not explored to generate ideas and information loses value. Consequently, questions arise about the cost-effectiveness of the whole data generation process and whether it is a good use of effort. The DHIS2 laboratory data has the potential to generate information, identify diagnostic gaps, determine the extent of coverage, and track intervention progress [24]. To achieve this, both national and sub-national data quality monitoring is key to ensure that the monthly laboratory data are reported accurately, in a timely and consistent fashion by all eligible facilities to support evidence-based decision making.

Our analyses revealed poor reporting consistency of the DHIS2 laboratory testing activities. Only 41.3% (74/179) of the reporting facilities submitted their laboratory testing activities consistently for the 24 months, this being 36.3% (74/204) of the total facilities. The remainder of the facilities either failed to submit their laboratory data in DHIS2 completely (12.3%, n = 25/204) or partially reported in some of the 24 months (51.5%, n = 105/204). Maina et al. also observed a similar poor reporting trend of malaria diagnostic activities reported in DHIS2 across public hospitals in Kenya [23]. We did not investigate the main reasons behind the poor reporting of diagnostic activities in DHIS2, but it is notable that no reports were received from two tertiary public hospitals and several large private facilities, all of which might offer a wider array of diagnostic tests. There are other programs besides DHIS2 where clinical laboratories also provide testing information such as for tuberculosis (TB) and HIV [24, 25], this may result in parallel reporting and undermine wider reporting. Laboratory managers and HRIOs concerned with DHIS2 laboratory data might be encouraged to submit data if systems of routine feedback from their national and county supervisors with regards to quality of data submitted are in place. However, our experience suggests this is not the case and likely undermines laboratory data reporting in DHIS2.

The main limitation of our analysis was that we were unable to differentiate between facilities that failed to submit testing reports due to lack of testing capacity and those that did not report because no patients needed the tests or simply no test done. This challenge was brought about by the fact that DHIS2 converts zero values to missing data (‘N/A’), an observation that was also noted elsewhere [19]. Ideally, one would expect a facility without capacity to conduct certain tests to have missing reports (‘N/A’) while tests not done because they weren’t requested would be denoted by zero test volumes. However, this was not the case because the majority of the zero test volumes and missing reports were all denoted as missing values by the DHIS2 system. To mitigate this, we assumed that all the missing values were zeros and therefore, a facility had the capacity to conduct a given test if it ever reported a non-zero test volume for the same test in any of the 24 months. Hospitals that did not report conducting a particular test for all the 24 months examined were interpreted as having no capacity to conduct the test.

The data suggest frequent test unavailability within county hospitals in Kenya that serve populations of 100,000 to 1,000,000 [16]. Many hospitals seem to have much lower diagnostic capacity than policy and strategy documents recommend [26]. The overall testing scope among the studied hospitals was low (40%). Also, the testing capacity increased with an increase in bed capacity. This suggests that higher tier hospitals as indicated by bed capacity are likely to offer a wider scope of tests. This finding concurs with Yadav et al. who also noticed that test availability increased in higher hospital tiers within ten LMICs including Kenya. However, the overall testing scope of 40% as observed in our analysis was slightly lower compared to the 55.5% reported by Yadav et al [12]. This difference can be explained in two dimensions; firstly, the poor reporting of diagnostic testing in Kenya as noted in our findings and secondly, the fact that our analysis included a broader scope of tests as compared to the few essential tests analysed in the other study. Although some bigger hospital laboratories offer a relatively higher scope of tests, we believe that the variations observed in the number of test types reported every month within hospitals point to frequent test unavailability across facilities. This pattern is also noted among a specific set of WHO EDL tests, with some hospitals unable to offer any of the 46 EDL tests for more than six consecutive months. Ward et al. also reported frequent unavailability of EDL tests within clinical laboratories in Ghana [10].

Unlike bigger hospitals, smaller hospitals (less than 100-bed capacity) had a lot of variations in their testing scope. This can be explained by the fact that most private facilities (79%, 27/34) assessed fall in this category. Further stratification of testing scope by hospital ownership confirmed the testing scope variations in private hospitals. Although private laboratories are required to meet a certain testing scope threshold by the government during registration and licensing, this only applies to a few tests depending on the laboratory’s size. Generally, the testing scopes in the private sector rely on their financial capabilities, prioritization and the need to remain competitive in the diagnostic market. This could explain why we observed a bigger testing scope in private hospitals as compared to public hospitals. All in all, the overall fluctuation in test availability and the limited testing scopes show the challenges experienced by patients in need of differential diagnosis, especially in public facilities. Under such circumstances, patients may need to move from one clinical laboratory to another in search of laboratory tests [27]. Such movements are costly, time consuming for the patients and unnecessarily increase the turnaround time, consequently resulting in delayed care [28].

The majority of hospitals reporting data in DHIS2 do not report on anatomical pathology and cancer tests. Save for PSA test, more than 70% of the hospitals neither reported anatomical pathology nor cancer testing activities. We noted that in four (4/47) counties their hospitals did not report any cancer or anatomical pathology tests for the entire 24 months. There is no doubt that anatomical pathology and cancer tests are in very high demand especially with the current surge of cancer cases in Kenya [29]. Failure to report on these tests is an indication that many general hospitals in Kenya lack appropriate diagnostic capacity in this area [29] and consequently healthcare providers may resort to using a ‘working diagnosis’ [26] even for cancer care, which will critically undermine their ability to offer quality care. The alternative for health care providers in public hospitals is to refer patients for laboratory diagnosis from private facilities or private standalone laboratories. However, most of these private facilities and standalone laboratories are found in larger towns and cities and the cost of diagnostic tests may be unaffordable to the majority of the patients [30].

Clinical laboratories in LMICs experience several problems that contribute to test unavailability. Issues of inadequate supply of laboratory consumables and frequent breakdown of laboratory equipment are common in clinical laboratories in Kenya [2, 26]. This may explain the testing inconsistency and the frequent test unavailability noted in the current analysis. Similarly, lack of proper diagnostic equipment, insufficient human resource and expertise pose a great challenge to the availability of certain tests such as anatomical pathology and cancer. According to Wilson et al. tests of anatomical pathology may require up to three personnel; a histotechnologist to process tissues, a cytotechnologist to process fluids and a pathologist to examine the slides and make a diagnosis [1]. Most general hospitals in Kenya do not have qualified full-time pathologists, making anatomical pathology testing almost unachievable [31]. Unfortunately, save for donor funded programs such as HIV and TB, most of the laboratories in Kenyan public hospitals do not have systems in place to support specimen referral. Telepathology has shown good prospect in anatomical pathology especially in LMICs where qualified pathologists are few, but its implementation is costly in terms of the required staff-time, equipment and bandwidth [2, 32, 33]. Equally, such hospitals may revert to the detection of biomarkers for cancer diagnosis, but this method relies on high technology equipment and consumables which may also not be available [33].

### Limitations

Our analysis has some limitations. Our inclusion criteria of general hospitals may have resulted in us missing hospitals perhaps especially in the private sector although missing larger hospitals is less likely. Notably, none of the tertiary hospitals in our sample and three large private hospitals reported their laboratory testing data in DHIS2. Therefore, the laboratory testing capacity of these better equipped facilities likely to offer a wider scope of tests was not evaluated. Perhaps more importantly, our dataset does not include any data from standalone laboratories that are an increasingly common feature of the private health care sector in Kenya. Our findings of poor availability of diagnostic laboratory services may therefore be most applicable to those from low-income households and those from more rural counties, populations that may rely much more on the lower cost, more geographically accessible public sector. Separately, we acknowledge that the classification of the analysed tests into common and uncommon tests by the laboratory managers may not be a universally accepted standard way of classification. Nonetheless, the classification is supported by our data given that the common tests were indeed more likely to be available. Also, since the DHIS2 system converts zero events entered to missing data (‘N/A’), we assumed that all the values indicated as missing were zeros. This may not always be the case as there are instances where tests were done but the data were not entered. In which case our results may slightly underestimate the availability of diagnostic capacity although we typically considered any report of non-zero activity in any one of the 24 months as an indication of test availability.

## Conclusion

The current reporting of laboratory testing information in DHIS2 remains poor with several large private facilities and tertiary hospitals failing to submit their monthly laboratory testing reports. Monitoring of heterogeneity in laboratory testing accessibility across the country would require significant improvements in consistency and coverage of routine laboratory test reporting in DHIS2. Nonetheless, current data suggest considerable fluctuations in the scope of MoH recommended and WHO EDL tests that likely reflect inadequacies in supplies, expertise and equipment failures. Data further suggest limited access to diagnostic testing at hospital level that would support diagnosis of many chronic and non-communicable diseases and even common cancers in Kenya.

## Supporting information

S1 Table Shows all the tests included in our analyses. The tests are reported by hospital laboratories in DHIS2 every month.
(DOCX)
