# Supplementary material for: Reporting of diagnostic and laboratory tests by general hospitals as an indication of access to diagnostic laboratory services in Kenya
Source: PLoS One. 2022 Apr 8;17(4):e0266667. doi: 10.1371/journal.pone.0266667 (PMC8992978; doi:10.1371/journal.pone.0266667)
Supplement: S1 Table — The tests are reported by hospital laboratories in DHIS2 every month. (DOCX) [file pone.0266667.s001.docx]

**Appendix 1: The total number of tests analysed in EDL categories (N=80).**

| **Number** | **EDL test category** | **Tests** | **EDL test** | **Type** |
| --- | --- | --- | --- | --- |
| 1 | Sexually Transmitted | EID | Yes | Common |
| 2 |  | HIV | Yes | Common |
| 3 |  | TPHA | Yes | Uncommon |
| 4 |  | VDRL | Yes | Common |
| 5 |  | Viral load | No | Common |
| 6 | Haematology | Blood group and crossmatch | Yes | Common |
| 7 |  | CD4 count | Yes | Common |
| 8 |  | Coagulation profile | Yes | Uncommon |
| 9 |  | ESR | Yes | Common |
| 10 |  | Full blood count | Yes | Common |
| 11 |  | HB estimation | Yes | Common |
| 12 |  | Peripheral blood films | Yes | Common |
| 13 |  | Reticulocyte Count | No | Uncommon |
| 14 | Clinical Chemistry | Blood Sugar | Yes | Common |
| 15 |  | CSF Glucose | Yes | Common |
| 16 |  | CSF Proteins | Yes | Common |
| 17 |  | HCG | Yes | Common |
| 18 |  | Liver Function Tests | Yes | Common |
| 19 |  | Low Density Lipids | Yes | Common |
| 20 |  | OGTT | Yes | Uncommon |
| 21 |  | Renal Function Tests | Yes | Common |
| 22 |  | Rheumatoid factor | No | Common |
| 23 |  | Sickling test | Yes | Common |
| 24 |  | T3 | No | Uncommon |
| 25 |  | T4 | No | Uncommon |
| 26 |  | TSH | Yes | Common |
| 27 |  | Total cholesterol | Yes | Common |
| 28 |  | Triglycerides | Yes | Common |
| 29 |  | Urine Chemistry | Yes | Common |
| 30 | Cancer | CA 15-3 | No | Uncommon |
| 31 |  | CEA | No | Uncommon |
| 32 |  | PSA | Yes | Common |
| 33 | Bacteriology, Mycology and Parasitology | ASOT | No | Common |
| 34 |  | B. anthracis | No | Uncommon |
| 35 |  | Bacterial Meningitis (CSF) | Yes | Common |
| 36 |  | Blood bacteriology | Yes | Uncommon |
| 37 |  | Brucella | No | Common |
| 38 |  | CRAG Test | Yes | Common |
| 39 |  | H. pylori | Yes | Common |
| 40 |  | Hepatitis A test | Yes | Common |
| 41 |  | Hepatitis B test | Yes | Common |
| 42 |  | Hepatitis C test | Yes | Common |
| **Number** |  | **Tests** | **EDL test** | **Type** |
| 43 |  | High Vaginal Swab | Yes | Common |
| 44 |  | MDR_TB | Yes | Common |
| 45 |  | Malaria BS | Yes | Common |
| 46 |  | Malaria RDT | Yes | Common |
| 47 |  | Pus swabs | Yes | Common |
| 48 |  | Rectal swabs | Yes | Uncommon |
| 49 |  | Stool Examination | Yes | Common |
| 50 |  | Stool culture | Yes | Uncommon |
| 51 |  | TB Culture | Yes | Common |
| 52 |  | TB Sputum smears | Yes | Common |
| 53 |  | Throat swab bacteriology | Yes | Uncommon |
| 54 |  | Urethral swabs | Yes | Uncommon |
| 55 |  | Urine Microscopy | Yes | Common |
| 56 |  | Urine bacteriology | Yes | Common |
| 57 | Anatomical pathology | Ascitic fluid cytology | - | Uncommon |
| 58 |  | Bone marrow aspirate | - | Common |
| 59 |  | Breast FNA | - | Common |
| 60 |  | Breast tissue histology | - | Uncommon |
| 61 |  | CSF cytology | - | Uncommon |
| 62 |  | Cervix tissue histology | - | Uncommon |
| 63 |  | Dental tissue histology | - | Uncommon |
| 64 |  | GIT tissue histology | - | Uncommon |
| 65 |  | Head and Neck tissue histology | - | Uncommon |
| 66 |  | Liver FNA | - | Uncommon |
| 67 |  | Lymph nodes FNA | - | Uncommon |
| 68 |  | Lymph nodes tissue histology | - | Uncommon |
| 69 |  | Ovary tissue histology | - | Uncommon |
| 70 |  | PAP smear | - | Common |
| 71 |  | Pleural fluid cytology | - | Common |
| 72 |  | Prostate tissue histology | - | Uncommon |
| 73 |  | Skin tissue histology | - | Uncommon |
| 74 |  | Soft tissue masses FNA | - | Common |
| 75 |  | Thyroid FNA | - | Uncommon |
| 76 |  | Tissue impressions | - | Uncommon |
| 77 |  | Touch preparations | - | Uncommon |
| 78 |  | Trephine biopsy | - | Uncommon |
| 79 |  | Urine cytology | - | Uncommon |
| 80 |  | Uterus tissue histology | - | Uncommon |
